# White matter hyperintensity burden and collateral circulation in acute ischemic stroke with large artery occlusion

**DOI:** 10.1186/s12883-023-03517-8

**Published:** 2024-01-02

**Authors:** Wang Chen, Meihong Wang, Lei Yang, Xianjun Wang, Qianxiu Jin, Zhenyu Zhao, Wenli Hu

**Affiliations:** 1 Department of Neurology, Beijing Chaoyang Hospital, Capital Medical University, No. 8 Gongti South Road, Chaoyang, Beijing, 100020 China; 2 Department of Neurology, Yishui People’s Hospital, Linyi, Shandong China; 3 Department of Neurology, Linyi People’s Hospital, No. 27, Crossroads with Wuhan and Wohushan St, Linyi, 276000 Shandong China; 4 Department of Imaging, Linyi People’s Hospital, Linyi, Shandong China

**Keywords:** White matter hyperintensity, Collateral circulation, Acute ischemic stroke, Large vessel occlusion, Thrombectomy

## Abstract

**Objective:**

This study aimed to investigate the association between white matter hyperintensity (WMH) burden and pial collaterals in acute strokes caused by intracranial large artery occlusion treated with mechanical thrombectomy in the anterior circulation, focusing on stroke subtypes.

**Methods:**

Consecutive patients undergoing mechanical thrombectomy between December 2019 and June 2022 were retrospectively screened. The Fazekas scale assessed WMH burden. Pial collaterals were categorized as either poor (0–2) or good (3–4) based on the Higashida score. A multivariable analysis was used to determine the relationship between WMH burden and pial collaterals. Subgroup analyses delved into associations stratified by stroke subtypes, namely cardioembolism (CE), tandem lesions (TLs), and intracranial atherosclerosis (ICAS).

**Results:**

Of the 573 patients included, 274 (47.8%) demonstrated poor pial collaterals. Multivariable regression indicated a strong association between extensive WMH burden (Fazekas score of 3–6) and poor collaterals [adjusted OR 3.04, 95% CI 1.70–5.46, *P* < 0.001]. Additional independent predictors of poor collaterals encompassed ICAS-related occlusion (aOR 0.26, 95% CI 0.09–0.76, *P* = 0.014), female sex (aOR 0.63, 95% CI 0.41–0.96, *P* = 0.031), and baseline Alberta Stroke Program Early Computed Tomography scores (aOR 0.80, 95% CI 0.74–0.88, *P* < 0.001). Notably, an interaction between extensive WMH burden and stroke subtypes was observed in predicting poor collaterals (*P* = 0.001), being pronounced for CE (adjusted OR 2.30, 95% CI 1.21–4.37) and TLs (adjusted OR 5.09, 95% CI 2.32–11.16), but was absent in ICAS (adjusted OR 1.24, 95% CI 0.65–2.36).

**Conclusions:**

Among patients treated with mechanical thrombectomy for anterior circulation large artery occlusion, extensive WMH burden correlates with poor pial collaterals in embolic occlusion cases (CE and TLs), but not in ICAS-related occlusion.

## Introduction

Mechanical thrombectomy (MT) has emerged as the primary treatment approach for patients with anterior circulation stroke caused by intracranial large artery occlusion [1]. The role of collateral circulation, especially pial collaterals, is pivotal in predicting arterial recanalization, hemorrhagic transformation, and outcomes following MT [2–5]. Given that pial collaterals are anatomically part of the microvasculature circulation, investigating the relationship between cerebral small vessel disease and pial collaterals is both innovative and logical.

White matter hyperintensity (WMH), presumed to be of vascular origin and manifested as small vessel disease on magnetic resonance imaging (MRI), is commonly associated with age and vascular risk factors [6]. Although the precise pathogenesis of WMH is largely undetermined, it is suggested that WMH reflects blood–brain barrier vulnerability and impaired vascular autoregulation resulting from microvascular endothelial dysfunction [7]. Such impaired vascular autoregulation might potentially influence the status of pial collaterals [8]. Recent studies have yielded conflicting findings regarding the association of WMH burden with pial collaterals in patients undergoing MT for intracranial large artery occlusion [9–14]. However, research specifically exploring this relationship with a focus on stroke etiology, especially in the context of intracranial atherosclerosis (ICAS)-related large vessel occlusion and its impact on WMH burden and pial collaterals, remains notably absent.

In China, strokes attributed to intracranial atherosclerosis (ICAS) have been reported to have a prevalence as high as 46.6% [15]. ICAS exhibits distinctions from embolism. A previous study explored the relationship between WMH burden and pial collaterals amidst cardioembolic and artery-to-artery embolic large artery occlusion [11]. We aimed to explore the association of WMH burden with pial collaterals based on cardioembolic, and atheroembolic, as well as ICAS-related stroke in patients treated with EVT for anterior circulation.

## Methods

### Study population

We systematically reviewed a continuous cohort of patients diagnosed with acute ischemic stroke due to large artery occlusion. These patients underwent emergency digital subtraction angiography (DSA) and were drawn from the Linyi People’s Hospital database, spanning December 2019 to June 2022. Patients eligible for our study received anterior circulation MT within 24 h post-symptom onset and had either a pre-treatment or post-treatment MRI conducted within five days. Exclusion criteria included ineffective evaluation of pial collaterals on DSA, absence of T2-weighted fluid-attenuated inversion recovery (FLAIR) images, isolated occlusion of the extracranial carotid artery, anterior cerebral artery occlusion, and bilateral occlusion. The research was conducted in line with the Declaration of Helsinki and adhered to the STROBE guidelines [16]. Both Beijing Chaoyang Hospital and Linyi People’s Hospital’s institutional ethics committees granted ethical approval for this study. Due to its retrospective design and the anonymization of data, the ethics committee of Linyi People’s Hospital waived the requirement for informed consent.

Data regarding demographics, medical history, imaging, laboratory findings, and baseline clinical characteristics were collected. Baseline symptoms of neurologic deficits were assessed using the National Institutes of Health Stroke Scale (NIHSS) scores [17]. Early ischemic alterations were identified on baseline imaging, as documented by the Alberta Stroke Program Early CT Score (ASPECTS) [18]. ICAS-related occlusion was recognized when stenosis exceeded 70% or when stenosis was greater than 50% coupled with indications of distal blood flow disturbance or evidence of recurrent re-occlusion after thrombus removal during MT [19]. Tandem lesions (TLs) were identified by the presence of intracranial embolus originating from extracranial atherosclerotic occlusion of the internal carotid artery. Carotid atherosclerosis was identified through retrospective analysis of DSA imaging, which involved detecting calcifications and examining ‘slim waist’ morphology during balloon dilatation procedures [20]. Additionally, post-endovascular treatment carotid artery ultrasound was used to assess ruptured plaques. The determination of occlusion associated with cardioembolism (CE) was based on a standard that incorporates both high and low embolic potentials, as recommended [21]. This included post-treatment evaluations, such as identifying atrial fibrillation, to assess embolic potential. Other subtypes of large artery occlusion included dissection, vasculitis, and other well-defined cause, as well as cases with uncertain etiology. Stroke etiology was evaluated by a neuro-interventionist (W.C.) who was uninformed about patients’ clinical and MRI details. When etiology determination posed challenges, a senior neuro-interventionist (Z.Z.) was consulted for resolution.

### Assessment of white matter hyperintensity

WMH was primarily attributed to vascular origins in this study. To maintain focus, other potential causes of WMH, such as metabolic disorders or demyelination, were excluded from our analysis. WMH was assessed using a 3.0 Tesla MRI scanner (Siemens, Verio, Germany), with hyperintense areas on T2-weighted FLAIR images being identified as WMH, consistent with vascular etiology [6]. To distinguish acute stroke hyperintensities from chronic WMH on these images, Diffusion-Weighted Imaging (DWI) was utilized; regions positive in FLAIR and negative in DWI were classified as WMH. The Fazekas scale was implemented to grade the WMH burden [22]. The periventricular hyperintensity was visually scored as: 0 (absent), 1 (cap or pencil lining), 2 (smooth halo), and 3 (irregular periventricular hyperintensity extending into the deep white matter). Meanwhile, deep hyperintensity was scored as: 0 (absent), 1 (punctate foci), 2 (beginning confluence of foci), and 3 (large confluent areas) [22]. The scores for periventricular and deep hyperintensity were summed to compute a total Fazekas score, ranging from 0 to 6 [23, 24]. WMH was assessed in both hemispheres, recording the highest degree of burden. The evaluation of WMH burden was independently conducted by a neuroradiologist (Q.J.) and a neurologist (M.W.), both blinded to the patients’ clinical and angiographic data. Any discrepancies were resolved through discussion between the two assessors. The inter-rater reliability for periventricular and deep WMH burden was assessed using the kappa statistic, yielding values of 0.86 and 0.83, respectively.

### Assessment of pial collateral circulation

Pial collaterals were evaluated using DSA and categorized according to the Higashida score [25]. This evaluation entailed angiographic analysis of both the ipsilateral and contralateral internal carotid arteries, along with the vertebral artery, conducted before the thrombectomy procedure. The score interprets the grades as follows: 0 = no visible collaterals in the ischemic territory, 1 = slow collaterals in the peripheral ischemic territory with persistent defect, 2 = fast collaterals in the peripheral ischemic territory with persistent defect, 3 = slow collaterals with complete blood flow within the ischemic territory in the late venous phase, and 4 = fast collaterals with complete blood flow throughout the ischemic territory via retrograde filling. Pial collaterals were dichotomized as either poor (grades 0–2) or good (grades 3–4). The collateral grading was scrutinized and validated by a board-certified neurologist (X.W.) and a neuro-interventionist (W.C.), with any disparities in readings being settled through discussion. To quantify the reliability of these evaluations, we calculated the kappa values for interrater agreement, which demonstrated a high level of concordance (kappa = 0.81) for categorizing collaterals as good or poor.

### Laboratory findings

A routine blood test was conducted prior to the endovascular treatment. Parameters such as Mean Platelet Volume (MPV), Platelet Distribution Width (PDW), and Red Blood Cell Distribution Width (RDW) were recorded. Specifically, RDW is a measure of the variation in red blood cell size or volume, which can reflect a range of health conditions. An elevated RDW, indicating notable variability in red blood cell size, has been linked to decreased red blood cell deformability, which might adversely affect microvascular perfusion in brain tissues [26].

### Statistical analysis

To explore the relationship between WMH burden and pial collaterals, statistical analyses were conducted. Continuous variables that adhered to a normal distribution were expressed as mean ± standard deviation (SD), whereas those not normally distributed were communicated as the median with interquartile range (IQR). Categorical variables were displayed as counts and percentages.

For the initial univariate comparisons between poor and good pial collaterals, the chi-square or Fisher’s exact test was utilized for categorical variables, and the t-test or Mann–Whitney U test was employed for continuous variables. Covariates, such as age, sex, WMH burden, stroke etiology, and other pivotal factors influencing pial collaterals, which showed a *p*-value < 0.1, were incorporated into a multivariable logistic regression model to evaluate their associations with poor collaterals. A multicollinearity test was executed, considering variables with a variance inflation factor (VIF) below five as indicative of no collinearity.

Receiver operating characteristic (ROC) curve analysis was conducted to establish the optimal threshold of the total Fazekas score for predicting poor pial collaterals. With the identified threshold value, WMH burden was categorized into mild or extensive. Lastly, a subgroup analysis was conducted, focusing on stroke subtypes (CE, TLs, and ICAS) to delve into the association of WMH burden with pial collaterals. A *p*-value < 0.05 was set as the threshold for statistical significance. The analyses were executed using SPSS software (version 25.0, IBM, USA) and Stata (version 14.0, Stata Corp, USA).

## Results

**Fig. 1:**
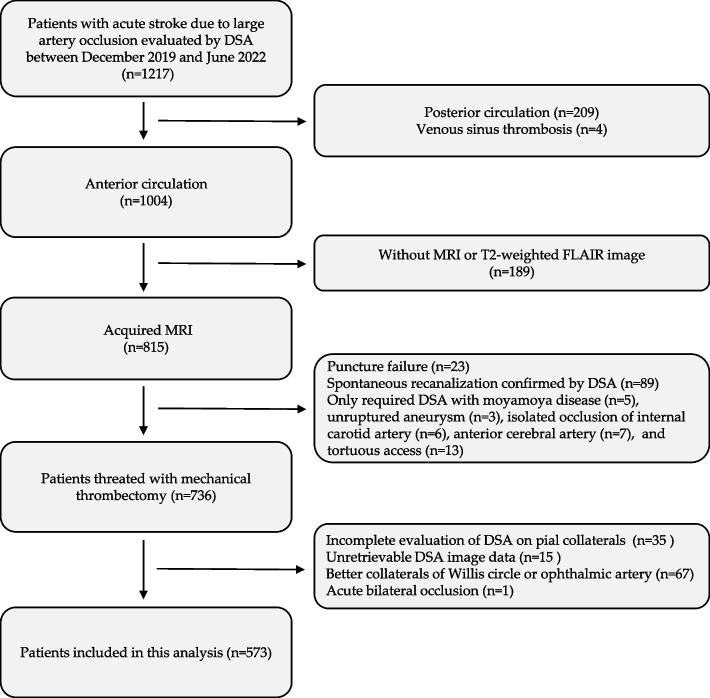
Flowchart of patient selection. DSA, digital subtraction angiography; MRI, magnetic resonance imaging; FLAIR, fluid-attenuated inversion recovery

Out of 1217 patients who underwent DSA, 573 patients (median age: 66 years; IQR: 57–72 years; 30.5% female) met the established inclusion criteria. The screening process, illustrated via a flowchart, can be viewed in Fig. 1. Comprehensive clinical, laboratory, and imaging characteristics of the participants, organized by pial collateral status, are detailed in Table 1.

**Table 1:** Participant characteristics dichotomized by pial collateral status

	**All patients** **(*n* = 573)**	**Poor collaterals** **(*n* = 274)**	**Good collaterals** **(*n* = 299)**	***p*-value**
**Demographics**				
Age, years, median (IQR)	66 (57–72)	65.5 (57–71)	66 (56–72)	0.817
Female, n (%)	175 (30.5)	68 (24.8)	107 (35.8)	0.004
**Medical history, n (%)**				
Hypertension	385 (67.2)	189 (69.0)	196 (65.6)	0.383
Diabetes	104 (18.2)	50 (18.2)	54 (18.1)	0.953
Dyslipidemia	183 (31.9)	95 (34.7)	88 (29.4)	0.179
Atrial fibrillation	162 (28.3)	84 (30.7)	78 (26.1)	0.225
Coronary heart disease	86 (15.0)	46 (16.8)	40 (13.4)	0.254
Current smoking	130 (22.7)	61 (22.3)	69 (23.1)	0.816
**Imaging features**				
Baseline ASPECTS, median (IQR)	7 (5–8)	6 (4–7)	7 (6–9)	< 0.001
Total Fazekas score, median (IQR)	2 (1–4)	3(1–4)	2(1–3)	< 0.001
Total Fazekas score, n (%)				0.003
Total Fazekas score 0	84(14.7)	30(10.9)	54(18.1)	
Total Fazekas score 1–2	227(39.6)	100(36.5)	127(42.5)	
Total Fazekas score 3–6	262(45.7)	144(52.6)	118(39.5)	
**Laboratory findings**				
HbA1c, %; median (IQR)^a^	5.9 (5.5–6.5)	5.8 (5.5–6.2)	5.9 (5.6–6.5)	0.210
Homocysteine, μ mol/L; median (IQR)^b^	11.4 (9.3–14.6)	11.2 (9–14.8)	11.7 (9.8–14.2)	0.698
RDW-SD, fL; median (IQR)	41.9 (40.3–43.8)	42.0 (40.4–44.2)	41.8 (40.2–43.4)	0.037
MPV, fL; mean ± SD	9.4 ± 0.9	9.4 ± 1.0	9.4 ± 0.8	0.945
PDW, CV%; median (IQR)	15.9 (12.9–16.2)	15.9 (12.8–16.2)	15.9 (13.7–16.2)	0.672
**Stroke etiology, n (%)**				< 0.001
Cardioembolism	236 (41.2)	119 (43.4)	117 (39.1)	
Intracranial atherosclerosis	189 (33.0)	64 (23.4)	125 (41.8)	
Tandem lesions	128 (22.3)	77 (28.1)	51 (17.1)	
Other stroke subtypes^c^	20 (3.5)	14 (5.1)	6 (2.0)	
**Occlusion site**				0.003
Intracranial internal carotid artery, n (%)	167 (29.1)	96 (35.0)	71(23.7)	
M1/M2, n (%)	406 (70.9)	178 (65)	228(76.3)	
**Clinical characteristics**				
Baseline NIHSS, mean ± SD	13.6 ± 5.9	14.7 ± 5.5	12.7 ± 6.0	< 0.001
Baseline SBP, mmHg; median (IQR)	155 (142–165)	155 (143–165)	155.3 (140–166)	0.555
Baseline DBP, mmHg; median (IQR)	87 (80–92.5)	88 (80–93)	87 (80–92)	0.885
Intravenous thrombolysis, n (%)	259 (45.2)	129 (47.1)	130 (43.5)	0.387
General anesthesia, n (%)	22 (3.8)	11 (4.0)	11 (3.7)	0.835
Onset-to-puncture time, min; median (IQR)	341 (219–495.5)	318.5 (217–454)	360 (225–570.5)	0.013
mTICI 2b/3, n (%)	526 (91.8)	246 (89.8)	280 (93.6)	0.092
sICH, n (%)	27 (4.7)	14 (5.1)	13 (4.3)	0.667

*ASPECTS* Alberta stroke program early computed tomography scores, *RDW-SD* red blood cell distribution width-standard deviation, *MPV* mean platelet volume, *PDW* platelet distribution width, *CV* variation coefficient, *M1/M2* first/second segment of the middle cerebral artery, *NIHSS* National institute of health stroke scale, *SBP* systolic blood pressure, *DBP* diastolic blood pressure, *mTICI* modified thrombolysis in cerebral infarction score, *sICH* symptomatic intracranial hemorrhage, *IQR* interquartile, *SD* standard deviation

^a^*n* = 363 included data

^b^*n* = 529 included data

^c^ including *n* = 10 for dissection, n = 9 for uncertain etiology, and n = 1 for ipsilateral occlusion of the carotid artery web with the middle cerebral artery

In this cohort, 274 patients (47.8%) displayed poor pial collaterals. Notably, both their distribution of total Fazekas scores and stroke subtypes exhibited significant disparities when compared with those exhibiting good collaterals (*P* = 0.003, *P* < 0.001, respectively). Furthermore, relative to patients with good collaterals, those with poor collaterals were characterized by a smaller proportion of females (*P* = 0.004), elevated incidence of intracranial carotid artery occlusions (*P* = 0.003), reduced baseline ASPECTS (*P* < 0.001), elevated baselineNIHSS scores (*P* < 0.001), increased RDW (*P* = 0.037), and a shorter onset-to-puncture time (*P* = 0.013).

Prior to the logistic regression model application, all pre-specified variables were verified to have VIF values under two, confirming no substantial collinearity. Post-covariate adjustment, a total Fazekas score of 3–6 (utilizing a total Fazekas score of 0 as reference) maintained a significant association with poor collaterals (aOR 3.04, 95% CI 1.70–5.46, *P* < 0.001). Additionally, correlations were noted between pial collaterals and variables such as sex (Female vs male, aOR 0.63, 95% CI 0.41–0.96, *P* = 0.031), stroke etiology of ICAS (over CE and TLs, with other subtype strokes as reference) (aOR 0.26, 95% CI 0.09–0.76, *P* = 0.014), and baseline ASPECTS (aOR 0.80, 95% CI 0.74–0.88, *P* < 0.001) (refer to Table 2). ROC analysis designated a total Fazekas score ≥ 3 as the optimal threshold for predicting poor pial collaterals, yielding an area under the curve of 0.589 (*P* < 0.001), with 0.53 sensitivity and 0.61 specificity. Consequently, WMH burden was categorized as mild (total Fazekas scores 1–2) and extensive (scores 3–6).

**Table 2:** Multivariate regression model for poor pial collaterals

	**Adjusted OR**	**95% CI**	***p*-value**
Age	0.986	0.968–1.004	0.137
Female	0.629	0.412–0.958	0.031
Baseline ASPECTS	0.804	0.738–0.875	< 0.001
Baseline NIHSS	1.031	0.995–1.067	0.090
Total Fazekas score 0	Reference	-	-
Total Fazekas score 1–2	1.732	0.984–3.050	0.057
Total Fazekas score 3–6	3.041	1.695–5.458	< 0.001
RDW	1.013	0.960–1.068	0.644
Cardioembolism	0.351	0.119–1.033	0.057
Intracranial atherosclerosis	0.260	0.089–0.761	0.014
Tandem lesions	0.605	0.202–1.812	0.369
Other stoke subtypes	Reference	-	-
Intracranial ICA occlusion	1.409	0.934–2.126	0.102
Onset-to-puncture time	1.000	0.999–1.000	0.253

*SPECTS* Alberta stroke program early computed tomography scores, *RDW* red blood cell distribution width, *NIHSS* National institute of health stroke scale, *ICA* internal carotid artery

Considering stroke subtypes among the 573 patients: 236 (41.2%) underwent MT for CE-related occlusion, 128 (22.3%) for TLs, and 189 (33%) for ICAS. A notable interaction between extensive WMH burden and stroke subtypes (*P* = 0.001) was observed in predicting poor collaterals. Extensive WMH burden was predictive of poor collaterals for CE (aOR 2.30, 95% CI 1.21–4.37) and TLs (aOR 5.09, 95% CI 2.32–11.16), but not for ICAS (aOR 1.24, 95% CI 0.65–2.36) (refer to Table 3).

**Table 3:** Subgroup analysis based on stroke subtypes for the association between extensive WMH burden and poor pial collaterals

	Adjusted OR^**a**^	95% CI	p (interaction)
			0.001
Extensive WMH burden (Cardioembolism)	2.301	1.213–4.368	
Extensive WMH burden (Intracranial atherosclerosis)	1.238	0.649–2.361	
Extensive WMH burden (Tandem lesions)	5.088	2.320–11.158	

^a^Adjusted for age, sex, baseline ASPECTS (Alberta stroke program early computed tomography scores), baseline NIHSS (National institute of health stroke scale), RDW (red blood cell distribution width), intracranial internal carotid artery occlusion, and onset-to-puncture time. WMH, white matter hyperintensity

## Discussion

This study illuminated a significant association between the extensive WMH burden and poor pial collaterals in patients undergoing MT for acute ischemic stroke due to a large artery occlusion in the anterior circulation. A 3.04-fold elevated risk of poor collaterals was identified in the presence of an extensive WMH burden as compared to their absence. However, the WMH burden did not correlate with the enlistment of pial collaterals in patients exhibiting an ICAS-related occlusion. Specifically, the influence of WMH burden on pial collateral recruitment was associated with sudden embolic occlusion, observed in both cardioembolism and atheroembolism, contrasted with occlusion originating from severe intracranial stenosis at the site.

A recent meta-analysis, incorporating data until August 2021, provided evidence supporting the predictive value of a high WMH burden for poor pial collaterals in patients undergoing MT [27]. Despite the notable heterogeneity in the findings, with various nations and WMH assessment methods under consideration, two French research teams opted for WMH volume as a meticulous measurement method to explore its association with pial collaterals. The outcomes from these teams were disparate; one elucidated a significant relationship [13], while the other disclosed no apparent correlation [14]. Noteworthily, both teams excluded patients with TL-related occlusions and did not include data on other stroke subtypes in their publications [13, 14]. Moreover, a Chinese study, inclusive of 78.2% of patients with ICAS-related occlusion, unveiled no marked correlation between the WMH burden and pial collateral circulation [10]. A pioneering study by Hashimoto (2022) established a correlation between the WMH burden and pial collaterals in individuals experiencing CE-related stroke [11]. Furthermore, our findings suggest that the predictive effect might be applicable to patients experiencing atheroembolism occlusion, as well as to those with CE-related occlusion.

Conversely, occlusion related to ICAS, as opposed to embolic occlusion, results in the gradual narrowing of the affected artery over time. This process typically induces a cerebral ischemia preconditioning state. When vascular stenosis reaches a threshold that instigates hemodynamic impairment, pial collaterals potentially activate as a principal blood supply conduit to preserve cerebral perfusion [28, 29]. Although the presumed vascular origin of WMH is linked to chronic microvasculature hypoperfusion, it is still ambiguous whether reduced cerebral blood flow is a precursor or a consequence of white matter lesions [30]. Additionally, as our research team has demonstrated, WMH presence signals blood–brain barrier dysfunction [31]. Furthermore, an extensive WMH burden might diminish cerebrovascular reactivity, indicating dynamic vascular dysfunction in cerebral blood flow autoregulation [32]. Compared to patients with embolic occlusion, those with ICAS-related occlusion and subsequent ischemic preconditioning establish inherent ischemic hemisphere protection [33]. In this context, the microvasculature’s reactivity and density, including pial collaterals, undergo remodeling and augmentation to offset cerebral hypoperfusion [33, 34], potentially presenting a neuroprotective effect that could retard the onset of WMH in the brain.

Theoretically, since cervical atherosclerosis can instigate chronic hypoperfusion, a favorable pial collateral condition would generally be anticipated in patients with TLs-related occlusion. While Guglielmi observed superior pial collaterals in strokes associated with TLs as opposed to those linked with CE causes [35], and Hassler reported similarly enhanced pial collateral status within TLs cohorts [36], Seeters found no association between TLs-related stroke and pial collateral status [37]. This might be attributed to the primary compensation by the circle of Willis against cerebral hypoperfusion due to carotid stenosis [37]. Our research corroborates Seeters’ findings, showing that patients with TLs-related occlusion did not demonstrate a correlation with favorable pial collaterals. In real-world scenarios, as atherosclerotic carotid stenosis progresses, both the circle of Willis and the ophthalmic artery act as compensatory mechanisms to mitigate cerebral hypoperfusion. Specifically, pial collaterals, while acting as a secondary reserve, play a less pronounced role in cerebral ischemia unless the entire circle of Willis is anatomically hypoplastic or absent [28]. Therefore, our study exposes a link between embolic occlusion and WMH burden, potentially due to the pial collaterals’ lack of preconditioning.

Echoing Seeters’ findings [37], our results did not demonstrate a significant association between vascular risk factors (such as age, hypertension, diabetes, dyslipidemia, and smoking) and the recruitment of pial collaterals. Interestingly, female patients in our study presented favorable pial collaterals, a finding that aligns with Eker et al. [12] and Forestier et al. [13] but deviates from the report by Derraz et al. [14], which documented unfavorable pial collaterals in female patients. The discrepancies related to sex might originate from various factors, including age, genetic predispositions, and sex hormone levels [38]. Nonetheless, these sex-based disparities did not influence post-MT outcomes and do not form crucial criteria for MT screening [39].

Our study has several strengths as it was the first to explore the relationship between WMH burden and pial collateral circulation in patients with embolic stroke (CE and TLs) as well as ICAS-related occlusion. This is of particular significance given the high prevalence of ICAS-related stroke, which can surge to 46.6% in China [15].

### Limitations

The conduct of this study at a single center introduces inherent limitations typically associated with retrospective studies. Our study incorporated patients who underwent MRI within five days of treatment. This approach is adopted due to the gradual progression of white matter lesions, which re-quires a considerable amount of time, and significant changes in WMH are unlikely to occur within a five-day period. Although WMH volume offers a more precise evaluation of WMH burden, the visual Fazekas scale may be more practical for assessing WMH burden in emergent patients in clinical settings. However, the absence of certain data, including missing MRI results, unretrievable DSA image data, and instances of puncture failure, may introduce selection bias into our study. This bias could potentially skew the study’s outcomes and interpretations. Consequently, future research, ideally in the form of multicenter studies, is required to validate our findings and to ensure their robustness and applicability in diverse clinical settings. Furthermore, future research targeting low-incidence stroke etiologies could yield essential insights into the variable impacts of WMH burden on pial collateral circulation within rarer stroke subtypes, including dissection and vasculitis.

## Conclusions

Extensive WMH is associated with poor pial collaterals in cases of embolic occlusion (CE and TLs) but not in ICAS-related occlusion, among patients undergoing MT for anterior circulation large artery occlusion. These findings imply that chronic injury to small vessels, coupled with the sudden onset of stroke in the absence of persistent hypoperfusion in large vessels, can potentially diminish the robustness of collateral circulation. Moreover, therapeutic interventions aimed at enhancing collateral circulation may be particularly beneficial in cases of severe small-vessel injury accompanying embolic occlusions in larger vessels.

## Data Availability

The data that support the findings of this study are available from the corresponding author upon reasonable request.

## Abbreviations

WMH: White matter hyperintensity
CE: Cardioembolism
TLs: Tandem lesions
ICAS: Intracranial atherosclerosis
MT: Mechanical thrombectomy
DSA: Digital subtraction angiography
VIF: Variance inflation factor
